# Modern Sociology

**Published:** 1898-11-26

**Authors:** 


### Modern Sociology.

### THE DIET OF PRISONERS.

By an Official.

The report of the Howard Association for the month of October deals with various of the provisions of the new Prisons Act, and we heartily agree with them in their strong appreciation of some of these, especially the Inebriates Act, which is admirable in every respect; but on the subject of prison dietary we cannot admit the justice of some opinions which they quote. The Committee, which at the present time is investigating this subject with the utmost care, will submit their conclusions to the Home Secretary, and it will be in his power and in that of all his successors, to make any alteration he pleases on the fare provided for convicted criminals, even to the extent, should such a consummation seem good to him, of rendering it absolutely luxurious. To such a fatal conclusion we trust no Home Secretary will ever come; but for the moment the controversy started by benevolent agitators rages round the question of what is called No. 1 Diet. Let us state, first, that this diet has already been substantially modified for all female prisoners, and practically no longer exists for them. It is quite probable, when the new Prisons Act takes effect after the new year, it will be found to have been altered also for men; but in the meantime what are the facts as regards this rule? We find the following passage in the report of the Howard Association: "Lord Leigh, who (with his friend Lord Norton) has long laboured for the best interests of the inmates, both of prisons and reformatories, has stated that he recently saw ten prisoners who could scarcely stand for want of proper food. They had been on short sentence, and had had No. 1 Diet, and they were unfit to do a day's work when they came out." We yield to none in grateful recognition of Lord Leigh's kindly benevolence towards a most unhappy class, and of the good work he has done on their behalf in regard to prisoners' aid societies and other matters, and, as there can be no question of his strict accuracy, we do not doubt for a moment that he did see ten men who appeared very weakly after having been in prison; but we contend that they must have been ill and feeble when they went into gaol—probably from having been out of work and nearly starving—as the plain facts of the case disprove the possibility of their having been reduced to that condition solely by the No. 1 class diet. Here is the exact truth respecting it: It applies only to the men whose sentence is for seven days, and not more. On the first day of their entrance to the prison they receive a full, substantial meal, and the No. 1 Diet begins only on the following day. As they are liberated early on the morning of the seventh day, with means for a good breakfast often given by someone connected with the prison, the time when they are subjected to this much-abused diet cannot exceed five days, and if a Sunday intervenes opportunely, it will be only four days. Now it is pathologically impossible that the wholesome, if meagre, food provided for that period could reduce any man to such a state of weakness that he could not stand, unless he were previously in a condition of great unhealthiness. One of the prison Governors who has been in office for many years, and who has studied this matter most carefully, declares emphatically that while he has seen many men, enfeebled by want, come in for a seven days' sentence and go out looking better and stronger, he has never known one who came in fairly robust and well who showed the smallest sign of having materially suffered by No. 1 Diet. An account was given to us lately of a prison in South Africa under Cape government and supervision, which affords a somewhat striking contrast to the English prison fare, concerning which our benevolent agitators are so deeply disquieted. It may be supposed that Boers and Zulus and Hottentots, though decidedly differing in colour, have as good appetites as our British convicts, yet this is their unvarying diet month after month, and, we conclude, year after year: A bowl of cooked mealies—which, the narrator stated, is the sort of food generally thrown to the pigs—is handed to them in the morning, and precisely the same unsavoury mess in the evening; and that is all, with the exception that twice in the week they are given one small piece of meat—no other change from year's end to year's end. Were they so much distressed by this diet as to long for night to bring sleep and oblivion of their sufferings, the provision made for their repose contrasts unfavourably with the plank bed of our friends at home, on which we happen to know many men can sleep very comfortably. Into the stone cell where the African prisoner is shut up till morning a single, not very thick or clean, blanket is flung, in which he rolls himself, without pillow or mattress of any kind, and that over his entirely naked body, though the nights are sometimes cold even in that climate. Yet our readers need not feel themselves moved to compassion for these dark-skinned criminals. The narrator who gave us the account stated that he had never seen a more cheerful and light-hearted body of men. Of course, their intelligence is not great; in mind and intellect they are somewhat like untrained children. Yet one might have expected this fact would only make them more tenacious of their bodily ease and more anxious for their creature comforts. But such is not the case; they make no complaint, and are merry and laughter-loving, and as they are allowed to herd together in the daytime they seem to enjoy themselves considerably. Of course, we do not mean in any way to compare our countrymen, however faulty, with those poor coloured prisoners; but does not the contrast of the systems show that there is a little over-refinement and false sentimentality in the agitation which is clamouring for every sort of alleviation to the punishment of criminals? It is a matter of more importance than might at first appear, that a right judgment on this subject should be general, for the apparent intention of pampering prisoners cannot but have a bad effect on the working classes—an honest man out of employment, with a large family to support, is constrained to envy the scoundrels who, through felony and burglary, are housed where daily wholesome meals are supplied to them with unfailing regularity.

